# Enlarged NT (≥3.5 mm) in the first trimester – not all chromosome aberrations can be detected by NIPT

**DOI:** 10.1186/s13039-016-0279-z

**Published:** 2016-09-07

**Authors:** Malgorzata I. Srebniak, Merel C. de Wit, Karin E. M. Diderich, Lutgarde C. P. Govaerts, Marieke Joosten, Maarten F. C. M. Knapen, Marnix J. Bos, Gerda A. G. Looye-Bruinsma, Mieke Koningen, Attie T. J. I. Go, Robert Jan H. Galjaard, Diane Van Opstal

**Affiliations:** 1 Department of Clinical Genetics, Erasmus Medical Center, Wytemaweg 80, 3015 CN Rotterdam, The Netherlands; 2 Department of Obstetrics and Gynecology, Erasmus Medical Center, Wytemaweg 80, 3015 CN Rotterdam, The Netherlands; 3 Foundation Prenatal Screening Southwest region of The Netherlands, Wytemaweg 80, Na-1509, 3015 CN Rotterdam, The Netherlands

**Keywords:** Array, Microdeletion, NIPT, Nuchal translucency, NT, Submicroscopic chromosomal abnormalities, Prenatal diagnosis

## Abstract

**Background:**

Since non-invasive prenatal testing (NIPT) in maternal blood became available, we evaluated which chromosome aberrations found in our cohort of fetuses with an enlarged NT in the first trimester of pregnancy (tested with SNP microarray) could be detected by NIPT as well.

**Method:**

362 fetuses were referred for cytogenetic testing due to an enlarged NT (≥3.5 mm). Chromosome aberrations were investigated using QF-PCR, karyotyping and whole genome SNP array.

**Results:**

After invasive testing a chromosomal abnormality was detected in 137/362 (38 %) fetuses. 100/362 (28 %) cases concerned trisomy 21, 18 or 13, 25/362 (7 %) an aneuploidy of sex chromosomes and 3/362 (0.8 %) triploidy. In 6/362 (1.6 %) a pathogenic structural unbalanced chromosome aberration was seen and in 3/362 (0.8 %) a susceptibility locus for neurodevelopmental disorders was found. We estimated that in 2–10 % of fetuses with enlarged NT a chromosome aberration would be missed by current NIPT approaches.

**Conclusion:**

Based on our cohort of fetuses with enlarged NT we may conclude that NIPT, depending on the approach, will miss chromosome aberrations in a significant percentage of pregnancies. Moreover all abnormal NIPT results require confirmatory studies with invasive testing, which will delay definitive diagnosis in ca. 30 % of patients. These figures are important for pretest counseling enabling pregnant women to make informed choices on the prenatal test. Larger cohorts of fetuses with an enlarged NT should be investigated to assess the additional diagnostic value of high resolution array testing for this indication.

## Background

Nuchal translucency (NT) measurement is widely used as a marker of fetal abnormalities both of chromosomal and non-chromosomal origin [1–3]. A fetal NT > 99th percentile is by definition found in about 1 % of pregnancies [4]. Enlarged NT is not only associated with aneuploidies and other chromosome abnormalities, but also with a number of genetic syndromes, as well as with structural congenital anomalies, mainly cardiac defects [3, 5, 6]. The majority of fetuses with NT ≥ 3.5 mm have a normal karyotype and the pregnancy outcome is highly dependent on the absence of anomalies on expert fetal ultrasound examination [7]. Since non-invasive prenatal testing (NIPT) in maternal blood became available, we evaluated which chromosome aberrations found in our cohort of fetuses with an enlarged NT in the first trimester of pregnancy (tested with SNP microarray) could be detected by NIPT as well. The results of this study can be used in pre-test counseling, which can be helpful for making informed choice between invasive and non-invasive genetic testing.

## Methods

362 women carrying a fetus with an enlarged NT (≥3.5 mm) in the first trimester were prospectively referred for Illumina SNP genotyping array as described before [8, 9]. Fetal material was obtained through chorionic villi sampling (311 cases) or amniocentesis (51 cases) after the NT was measured as a part of the first trimester combined screening or as part of the routine first trimester crown rump length (CRL) measurement for pregnancy dating. Samples collected in our central location and 3 satellite hospitals between 1st September 2011 until 31st March 2016 that were routinely referred for SNP array testing (0.15 Mb resolution) were included in this cohort. All cytogenetic tests were done in one central laboratory. This cohort overlaps slightly with the cohort published before [10]. To create a homogenous cohort as much as possible we excluded the following cases:

- fetuses referred for hydrops foetalis,
- fetuses referred for enlarged NT with co-existing congenital anomalies evident on the CRL scan or the NT scan

All samples were tested with QF-PCR or MLPA to detect common aneuploidies (rapid aneuploidy detection - RAD). When RAD detected trisomy 21 or 13, such samples were karyotyped (GTG banding analysis) to assess the recurrence risk. Cases of triploidy or trisomy 18 were not further tested. All cases showing normal RAD results or sex-chromosomal aneuploidy were tested with Illumina SNP array (HumanCytoSNP-12 or Infinium_CytoSNP_850K with analysis resolution of ca. 0.15 Mb) as described before [8].

To answer our research question we have divided chromosome aberrations in the following groups:

1. autosomal aneuploidies
2. sex-chromosome aneuploidies that may be detected by NIPT when sex-chromosomes analysis is included in the test
3. triploidy
4. pathogenic structural unbalanced chromosome aberrations (both microscopic and submicroscopic)
5. susceptibility loci for neurodevelopmental disorders.

Further, we evaluated which aberrations would theoretically be missed by current NIPT approaches (NIPT tests were not routinely performed in this cohort). To be able to make this assessment, for the purpose of this paper, we assumed that all non-mosaic aneuploidies (both autosomal and sex-chromosomal) would be detectable by current NIPT [11] as well as structural unbalanced aberrations larger than 10 Mb [12, 13]. We report estimated percentages of abnormal cases missed by particular NIPT approaches with Agresti–Coull (adjusted Wald) 95 % confidence intervals, which have higher coverage probability than large-sample Wald intervals, in particular for small proportions [14, 15].

## Results

**Fig. 1:**
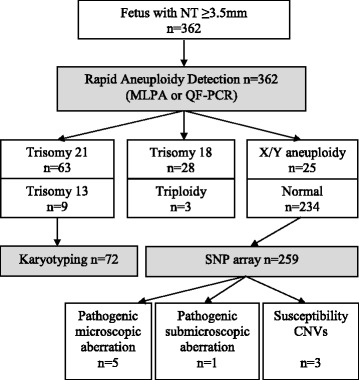
Diagnostic flow and the cytogenetic findings

The distribution of chromosomal abnormalities according to NT within the study population is presented in Table 1. The diagnostic flow was shown in Fig. 1. 38 % (137/362) of the cases showed abnormal cytogenetic results, which are presented in Table 2. The most common aberration was trisomy 21 (17 % 63/362) and in total in 28 % (100/362) of the cases an autosomal aneuploidy was detected (trisomy 21, 18 or 13). In 6/362 (1.6 %) a pathogenic unbalanced structural chromosome aberration was found: 5 were microscopically visible (>10 Mb) and 1 was submicroscopic (an atypical 22q11 microdeletion) [16]. There were three cases (3/362, 0.8 %) that showed a susceptibility locus for a neurodevelopmental disorder (2 cases of 15q11 microdeletion and one case of 16p11.2 microdeletion), these aberrations are probably not related to the enlarged NT.

**Table 1:** Distribution of chromosomal abnormalities according to NT within the study population (*n* = 362)

NT in mm	Number of cases in the cohort (%)	Number of cases with chromosome aberrations (% within the category)
3.5–4.4	179 (49 %)	35 (19 %)
4.5–5.4	68 (19 %)	32 (47 %)
5.5–6.4	42 (11.6 %)	30 (71 %)
6.5–7.4	24 (6.6 %)	14 (58.3 %)
7.5–8.4	14 (3.9 %)	6 (43 %)
≥8.5	10 (2.8 %)	6 (60 %)
unknown (hygroma colli, where NT measurement was not specified)	25 (7 %)	14 (56 %)
Total	362	137 (38 %)

**Table 2:** Results of cytogenetic testing in fetuses with enlarged NT (≥3.5 mm) or hygroma colli in the first trimester in fetuses referred for cytogenetic testing

Type of chromosome aberration	Number of fetuses *n* = 362	Potential detection by current NIPT approaches	% of anomalies that are going to be missed due to placental mosaicism or current testing resolution (>7–10 Mb)
Autosomal aneuploidy		Yes	3.5 % is likely to be missed [20, 49, 50] 0.8 % (3/362)
Trisomy 21	63 (17 %)		
Trisomy 18	28 (7.7 %)		
Trisomy 13	9 (2.5 %)		
Sex-chromosomal aneuploidy		Yes (if X/Y analysis are included)	10 % of monosomy X are likely to be missed [24] 0.5 % (2/362) Mosaic samples are most probably missed 0.5 % (2/362)
Monosomy X^a^	20 (5.5 %)		
XXX	2 (0.5 %)		
XXY	1 (0.3 %)		
Mosaic X/XY	2 (0.5 %)		
Triploidy	3 (0.8 %)	Yes (if SNP approach is applied)	Most of the current approaches cannot detect triploidy, and so far it is not possible to combine targeted SNP analysis with high coverage and whole genome profiling with high resolution 0.8 % (3/362)
Pathogenic unbalanced chromosome aberrations: 1) 46,XY,der [9] t (9;13) (q33.1;q12.11),-13 (NT 4.9 mm; 116 Mb gain at 9p24-q33)) 2) 46,XX,del [8] (p23.1) inv dup [8] (p11.21p23.1) (NT 4.6 mm; 28 Mb gain at 8p) 3) 45,XX,der [4] t (4;15) (q32.1;q13.3),-15dn (hygroma colli; 34 Mb loss at 4p)) 4) arr [hg19] 9p24.3p22.2 (46,587-18,277,618) x1 (NT 5.2 mm; 18 Mb loss at 9p) 5) arr [hg19] 1q32.1q44 (202,542,202-249,218,992) x3,9p24.3p24.1 (46,587-7,017,391) x1 (NT 3.6 mm, 47 Mb gain at 1q, 7 Mb loss 9p) 6) atypical 22q11 microdeletion of paternal origin arr [hg19] 22q11.21 (21,111,299-21,463,730) x1 pat (NT 4.3 mm, >0.5 Mb)	6 (1.6 %)	Larger than 10 Mb Yes (if whole genome profiling with resolution of 10 Mb is applied)	1/6 cases would be potentially missed: 0.3 % (1/362)
Susceptibility loci for neurodevelopmental disorders: 1) 15q11 microdeletion of NIPA1/NIPA2 of paternal origin arr [hg18] 15q11.2 (20,191,584-21,025,923) x1pat (NT 4.7 mm) 2) 15q11 microdeletion of NIPA1/NIPA2 of maternal origin arr [hg19] 15q11.2 (22,299,434-23,272,733) x1mat (NT 4.4 mm) 3) *de novo* 16q11.2 microdeletion arr [hg19] 16p11.2 (29,595,483-30,198,151) x1dn (hygroma colli)	3 (0.8 %)	No	So far genome wide detection of submicroscopic aberrations is not feasible. All will be missed 0.8 % (3/362)
Total number abnormal cases	137 (38 %)		

^a^One case showed also isochromosome Xp (case published before in [51])

Table 2 also shows the estimated percentage of cases that could be missed by current NIPT approaches in each category of chromosome aberrations. Based on our estimation a chromosomal anomaly would be undetected by NIPT in about 2–10 % of patients, depending on the approach used (Table 3).

**Table 3:** Percentage of abnormal cases missed by particular NIPT approaches. Additionally as 3.5 % of trisomy 13, 18 and 21 would be missed due to placental mosaicism 3/362 (0.8 %) cases were included in all NIPT options. As susceptibility CNVs are not the primary reason for invasive testing they were not included in the number of cases with structural anomalies that would be missed by NIPT

Type of NIPT testing	Percentage of patients in our cohort that would show false negative results with NIPT	95 % CI for percentage LL, UL	Number of cases that would be missed in our cohort
Targeted approach for trisomy 13, 18 and 21	10 % (37/362)	7.48, 13.80	34 + 3/362 (20x monosomy X, 5x other sex chromosomal aneuploidy, 3x triploidy, 6x structural anomalies + 3/362 (0.8 %) based on placental mosaicism)
Targeted approach for common aneuploidies of chromosomes 13, 18, 21, X/Y	4 % (14/362)	2.26, 6.44	11 + 3/362 (2x mosaic sex chromosomal aneuploidy, 3x triploidy, 6x structural anomalies + 3/362 (0.8 %) based on placental mosaicism)
Targeted approach for common aneuploidies of chromosomes 13, 18, 21, X/Y and triploidy	3 % (11/362)	1.64, 5.42	8 + 3/362 (2x mosaic sex chromosomal aneuploidy, 6x structural anomalies + 3/362 (0.8 %) based on placental mosaicism)
Whole genome profiling with a resolution of ca. 10 Mb	2 % (7/362)	0.09, 4.02	4 + 3/362 (3x triploidy and 1x submicroscopic anomaly will be missed + 3/362 (0.8 %) based on placental mosaicism)

*CI* confidence interval, *LL* lower limit, *UL* upper limit

## Discussion

Nuchal fluid accumulation may be caused by several factors, therefore, diverse genetic abnormalities may be expected in such fetuses. Taking this and numerous advantages of genomic microarrays [17] into account we have chosen a whole genome SNP array technique for cytogenetic investigations in cases of an enlarged NT [8, 9]. In the present study, we prospectively investigated the frequency of (sub) microscopic aberrations in this group of fetuses. Since non-invasive prenatal testing (NIPT) in maternal blood became available and the patients can face a choice between an invasive and non-invasive testing, we evaluated how high the risks of missing a pathogenic chromosome finding can be in a cohort of fetuses with enlarged NT measurement.

### Autosomal aneuploidies and triploidy

Although most of the anomalous fetuses showed trisomy 13, 18 or 21 (100/137, 73 %) and NIPT seems to be an excellent and safe test with a high positive predictive value in this selected high risk group, there are some drawbacks that have to be taken into account. One has to be aware of the risk of false-negative NIPT results for the common trisomies [18, 19]. 3.5 % of Down/Patau/Edwards syndrome cases would potentially be missed by NIPT due to generalized mosaicism with discrepant direct results (GMDD) [20]. Therefore, as shown in Table 2, at least 3 trisomic cases in the cohort presented in this paper (3.5 % out of 100 cases) would potentially be also missed if this cohort was tested with NIPT as a first-tier test. Additionally, other potential causes for false-negative NIPT results such as a low fetal DNA fraction (e.g. due to a high maternal body mass index) or technical failures, should also be taken into account [21]. The detection of triploidy is problematic as well. Although are SNP approaches are able to detect triploidy [22, 23], in our knowledge, there are no data showing that this could be achieved in assays based on whole genome shallow sequencing.

### Sex-chromosome aneuploidies

The second most common group of aberrations in fetuses with an enlarged NT is monosomy X (5.5 % 20/362, Table 2). Gil and colleagues showed that cfDNA tests could detect monosomy X in about 90 % of the cases [24], so at least 2 cases of monosomy X (10 % out of 20 cases) would be missed in this cohort. An accurate non-invasive detection of fetal monosomy X remains problematic due to several reasons. First of all, chromosomal mosaicism is common in sex chromosomal aneuploidy [25]*.* A low percentage of abnormal cells in the cytotrophoblast and co-existence of different abnormal cell lines may mask the actual chromosome aberration (e.g. 45,X/47,XXX) and lead to false negative results. There is also a maternal factor influencing the results as normal adult females show an age-related loss of X-chromosomes [26]. This mosaicism of chromosome X influences the positive predictive value in case of a monosomy X detection [27]. Moreover, finding sufficient Y-chromosome loci that are informative for copy number quantification may be difficult [28], causing monosomy X detection to be highly dependent on the NIPT method used. These difficulties are reflected in the recent literature that showed very limited follow-up of monosomy X cases diagnosed non-invasively [21, 29]. So although some authors suggest that there are accurate methods to detect fetal sex chromosomal aneuploidy in maternal plasma [30, 31], recent clinical experience shows low positive predictive value for monosomy X. This situation can be expected since the fetal DNA in maternal plasma is derived from the cytotrophoblast of chorionic villi (CV) and cytogenetic studies in CV already showed that sex-chromosomal aneuploidy in the cytotrophoblast of CV (STC-villi, short term cultured villi) is often not representative for the actual fetal karyotype [19, 32].

### Pathogenic unbalanced structural aberrations

Although an enlarged NT was observed in fetuses with unbalanced translocations, no significant differences were seen in a study that evaluated the role of nuchal translucency (NT) in the prediction of unbalanced translocation in offspring of couples carrying balanced translocations [33]. A recent study of Christiansen and colleagues showed that the distributions for NT measurements in case of an aberration other than trisomy 21, 13 or 18 more closely resembled that of the normal population [34]. So the detection of rare unbalanced chromosome anomalies in cohorts with an enlarged NT may be co-incidental. Nevertheless karyotypically visible unbalanced chromosome aberrations are likely to be detected by whole genome profiling NIPT approaches [12, 13, 35]. Unfortunately it is difficult to assess how many may be missed due to their mitotic origin and absence in the cytotrophoblast.

The incidence of pathogenic submicroscopic chromosomal abnormalities in fetuses with an enlarged NT has been studied by only few groups [36–41] resulting in conflicting conclusions [42, 43]. These differences in frequencies of chromosomal aberrations in published cohorts were observed before [42] and it may be a consequence of both cohort selection and differences in array design. Many of previously reported cohorts were either retrospectively tested and highly selected [38, 39] or included a heterogeneous group of fetuses with and without additional ultrasound anomalies detected in both first and second trimester [36, 38, 44, 45]. Our results show that the prevalence of submicroscopic aberrations in fetuses with enlarged NT resembles the prevalence in fetuses without ultrasound anomalies. This confirms previous results published by Huang and colleagues [39]. Therefore, in our opinion larger unselected cohorts with enlarged NT should be published to assess the actual risk of a pathogenic submicroscopic unbalanced chromosome aberration when an enlarged NT is diagnosed in the first trimester.

### Susceptibility loci for neurodevelopmental disorders

We did not take the susceptibility loci for neurodevelopmental disorders into account for the calculations shown in Table 3. Susceptibility CNVs are quite often found in prenatal array testing [46], however there is no study that showed any relationship with an enlarged NT. Finding additional predisposition factors may play a role in decision making in pregnancy, however it is less likely that one would choose invasive testing with a primary aim to investigate these. Therefore we assume that missing these findings is not the most important incentive for deciding on the prenatal test. Moreover the frequency of susceptibility CNVs in this cohort (0.8 % 3/362) resembles more the frequency in fetuses without ultrasound anomalies (1.3 %) [47] than those with ultrasound anomalies (2.7 %) [10].

## Conclusions

Since most (73 %; 100/137) chromosome aberrations in cases of an enlarged NT (≥3.5 mm) in the first trimester involved trisomy 21, 18 and 13, NIPT at first sight seems to be an appropriate test. However, our study confirms the previously published data by Lichtenbelt and colleagues [48] and shows that because of current limitations of NIPT (depending on the type of analysis) in ca. 2–10 % of cases with an enlarged NT a chromosome aberration will be missed by non-invasive testing. Moreover since the risk for a chromosome aberration is high (1:3) and since aberrant NIPT requires confirmatory studies due to the origin of the cfDNA, therefore NIPT as compared to invasive testing will delay a final diagnosis in about 30 % of patients. The limitations of NIPT should be clearly addressed in the pre-test counseling: possible diagnostic delay, the risk for false negative and false positive results and the fact that false-positive cases of monosomy X in enlarged NT fetuses may cause additional anxiety.

## Abbreviations

CNV: Copy number variation
CRL: Crown rump length
CV: Chorionic villi
DD: Developmental delay
GMDD: Generalized mosaicism with discrepant direct results
ID: Intellectual disability
MLPA: Multiplex ligation-dependent probe amplification
NIPT: Non-invasive prenatal test
NT: Nuchal translucency
QF-PCR: Quantitative fluorescence-polymerase chain reaction
RAD: Rapid aneuploidy detection
SL: Susceptibility locus
